# Emerging Material Paradigm: Strategic Optimization of Spinel Oxides as High-Performance Air Electrodes for Nanostructured Ceramic Fuel Cells

**DOI:** 10.3390/nano16030211

**Published:** 2026-02-06

**Authors:** Maoyi Hua, Lin Ge

**Affiliations:** College of Materials Science and Engineering, Nanjing Tech University, No. 30 South Puzhu Road, Nanjing 211816, China; 202461203210@njtech.edu.cn

**Keywords:** solid oxide fuel cells, proton ceramic fuel cells, air electrodes, spinel oxides

## Abstract

Hydrogen, renowned for its clean energy profile and high energy density, is a pivotal energy carrier for addressing global energy and environmental challenges. Solid oxide fuel cells (SOFCs) and proton ceramic fuel cells (PCFCs) have garnered significant interest due to their direct chemical-to-electrical-energy conversion, fuel flexibility, high efficiency, and environmental compatibility. However, conventional perovskite-based air electrodes suffer from sluggish oxygen reduction reaction (ORR) kinetics and insufficient structural stability at intermediate temperatures. Spinel oxides, distinguished by excellent chemical stability and thermal expansion compatibility, have emerged as promising alternatives; however, their broader application is constrained by their limited ionic conductivity and catalytic activity. This review systematically elucidates the crystal structure, intrinsic advantages, and advanced design strategies of spinel oxides. It particularly focuses on A- and B-site doping techniques for precise modulation of thermal expansion and enhancement of electrocatalytic performance, alongside high-entropy engineering approaches that bolster high-temperature stability. Finally, the review comprehensively discusses remaining challenges and future prospects for the implementation of spinel oxides in nanostructured ceramic fuel cells.

## 1. Introduction

In the global pursuit of clean and low-carbon energy systems, the reliance on conventional fossil fuels is increasingly challenged by their inherent inefficiencies and excessive pollutant emissions. This has underscored the urgent need for advancing high-efficiency and environmentally benign energy conversion technologies [1,2,3,4,5,6]. Ceramic fuel cells (CFCs) have emerged as one of the most promising sustainable energy technologies due to their high efficiency and fuel flexibility [7,8,9,10,11,12,13,14,15]. Typically, CFCs consist of three fundamental components: a porous air electrode, a dense electrolyte, and a porous fuel electrode. Based on the charge carriers and electrolytes, they are chiefly classified into solid oxide fuel cells (SOFCs) and proton ceramic fuel cells (PCFCs) [16,17]. SOFCs traditionally operate at elevated temperatures (typically >800 °C), which facilitates rapid electrochemical kinetics and supports fuel versatility, including natural gas and hydrocarbons, thereby broadening their practical applicability. Nonetheless, high-temperature operation poses challenges such as reduced longevity, increased manufacturing costs, and difficulties in sealing [18,19,20]. In contrast, PCFCs function efficiently at intermediate temperatures (500–700 °C), offering substantial promise owing to enhanced material stability under milder conditions [21,22,23,24,25]. Nevertheless, reducing the operating temperature inevitably leads to decreased electrical conductivity and diminished oxygen reduction reaction (ORR) activity at the air electrode, limiting overall cell performance [15,26]. Given the distinct advantages and intrinsic limitations of SOFCs and PCFCs, the development of novel air electrode materials with superior electrochemical properties is imperative to enhance conductivity and electrocatalytic efficiency [27,28,29,30,31,32,33,34,35,36,37,38].

**Figure 1:**
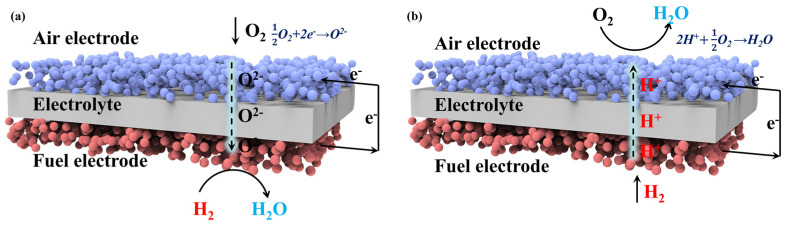
Schematic diagrams of working principles of (**a**) SOFCs and (**b**) PCFCs.

Although SOFCs and PCFCs share the fundamental fuel cells concept, their charge carriers and electrochemical reaction mechanisms are fundamentally distinct, leading to key differences in the operation of their air electrodes. SOFCs conduct oxygen ions (O^2−^) across the electrolyte. The overall reduction reaction at the air electrode can be expressed as: $\frac{1}{2}O_{2\;}+\;2e^{-}\;\to \;O^{2-}$ (Figure 1a), where oxygen molecules adsorb and dissociate on the electrode surface and are subsequently reduced by electrons to form O^2−^, which migrates through the electrode and electrolyte to the fuel electrode to react with fuel, producing H_2_O and releasing electrons.

In contrast, PCFCs rely on proton (H^+^) conduction. The overall air electrode reaction is $2H^{+}\;+\frac{1}{\;2}O_{2}\;\to {\;H}_{2}O$ (Figure 1b). In this system, H^+^ transport from the fuel electrode through a proton-conducting electrolyte to the air electrode, where oxygen undergoes adsorption, dissociation, and reduction, forming O^2−^ ions (or intermediate species). Water is then produced at the triple-phase boundary (TPB) by the recombination of H^+^ and O^2−^ species.

Despite differing charge carriers and sites of water formation, the air electrodes in both fuel cell types must satisfy several critical criteria to achieve high performance and durability: (I) High mixed ionic–electronic conductivity (O^2−^/e^−^ for SOFCs and H^+^/O^2−^/e^−^ in PCFCs) to facilitate charge transfer and enlarge active sites for ORR and oxygen evolution reaction (OER) [39,40], (II) sufficient porosity to enable efficient gas diffusion, and (III) excellent thermo-mechanical compatibility with electrolytes to prevent degradation or delamination during operation [41,42,43].

**Figure 2:**
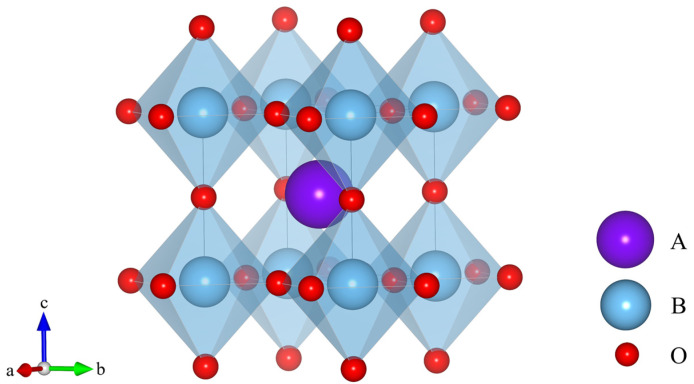
Representative structures of Perovskite-type oxide.

Over the past few decades, perovskite-type oxides have been predominantly employed as air electrode materials in SOFCs and PCFCs owing to their compositional versatility and tunable functional properties [44,45]. These complex oxides, generally expressed as ABO_3_ (where A is a lanthanide or alkaline earth metal, and B is a transition metal, such as Co, Fe, Mn, or Ni), feature a framework of corner-sharing BO_6_ octahedra that surround larger A-site cations (Figure 2). Their principal advantage lies in the capability for precise property tailoring via selective doping at the A- or B-sites (e.g., La, Sr, Co, Fe), enabling optimization of electrical conductivity, catalytic activity, and stability under various operational conditions.

However, perovskite proton conductors suffer from proton trapping phenomena, where acceptor dopants strongly bind protons via electrostatic interactions and the limited oxygen vacancy concentration hampers proton uptake, resulting in low proton conductivity at intermediate temperatures and constraining PCFC performance. Additionally, cobalt-based perovskites, while exhibiting excellent surface activity and electrical conductivity, often exhibit thermal expansion coefficient (TEC) mismatch with typical electrolytes, leading to internal stresses, interfacial delamination, or fractures during thermal cycling [46,47,48,49]. Moreover, the high cobalt content elevates material costs and exacerbates TEC mismatch. The segregation of Ba, Sr, and Co elements under prolonged high-temperature operation may also degrade electrocatalytic performance [50,51]. Compounding these issues, alkaline earth metals are prone to react with atmospheric CO_2_, forming non-conductive carbonates that impair long-term stability. As the operating temperature decreases, the conductivities and ORR activities of perovskite air electrodes become inadequate, thereby restricting cell power output. Additionally, sluggish ORR kinetics, which involve complex multistep electrochemical reactions requiring coordinated protons, oxygen ions, and electron transfer, remain a significant bottleneck, particularly reflected by higher polarization resistance (R_p_) in PCFC air electrodes relative to SOFCs. Collectively, these limitations have impeded the further deployment of perovskite oxides as air electrode materials.

In light of these challenges, this review shifts focus to a previously underexploited class of materials: spinel-type oxides as promising air electrodes in SOFCs and PCFCs, which have already found widespread application as protective coatings for interconnects in fuel cells [52,53]. Their distinctive crystal structures and favorable physicochemical properties hold potential to surmount the limitations of perovskites. Spinel oxides typically possess a high concentration of oxygen vacancies and exhibit excellent mixed ionic–electronic conductivity, which can enhance proton conductivity and ORR activity at intermediate temperatures, thereby mitigating performance constraints faced by perovskite counterparts under reduced-temperature operation. Furthermore, through compositional tuning, the thermal expansion coefficients of certain spinel oxides can be tailored to better match those of electrolytes used in SOFCs and PCFCs, thereby minimizing interfacial stresses and reducing the risk of mechanical failure. Notably, spinel-type oxides can achieve these benefits with low or no cobalt content, reducing costs and improving chemical stability by suppressing Ba and Sr segregation and carbonate formation upon exposure to CO_2_. Consequently, spinel oxides offer improved long-term operational stability. Importantly, when employed as air electrodes, these materials may deliver superior ORR kinetics and lower R_p_, paving the way for efficient, stable performance of SOFCs and PCFCs at intermediate temperatures.

## 2. Crystal Structure and Conduction Mechanism of Spinel Oxides

Spinel oxides, generally formulated as AB_2_O_4_, crystallize in the cubic system with the Fd-3m space group. Their distinctive crystal structure comprises a cubic close-packed oxygen ion framework, within which cations occupy two types of interstitial sites: tetrahedral (A-sites) and octahedral (B-sites). Each spinel unit cell contains 24 cations and 32 oxygen anions. Typically, A-site cations are generally divalent ions (e.g., Mg^2+^, Fe^2+^, Co^2+^, Ni^2+^, Mn^2+^, Zn^2+^, Cd^2+^), while B-site cations are trivalent (e.g., Al^3+^, Fe^3+^, Co^3+^, Cr^3+^, Ga^3+^). Notably, exceptions exist where A-site cations may exhibit tetravalent states (Ti^4+^, Sn^4+^, Zr^4+^) and B-site cations divalent states (Mg^2+^, Fe^2+^, Co^2+^, Ni^2+^, Zn^2+^) [54].

**Figure 3:**
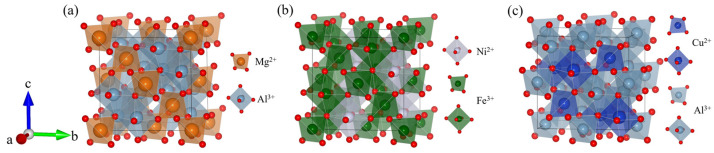
Representative structures of spinel: (**a**) a normal spinel (MgAl_2_O_4_), (**b**) an inverse spinel (NiFe_2_O_4_), and (**c**) a complex spinel (CuAl_2_O_4_).

Based on the cation distribution pattern, spinel structures can be broadly categorized into three types: normal spinel, inverse spinel, and complex spinel (Figure 3). For the normal spinel (AB_2_O_4_), A-site cations occupy tetrahedral sites, while B-site cations reside in octahedral sites; for the inverse spinel (B(AB)O_4_), all A-site cations occupy octahedral sites, while half of the B-site cations occupy tetrahedral sites, and the remaining half occupy octahedral sites; for the complex (or mixed) spinel (A_1−*x*_B*_x_*)(A*_x_*B_2−*x*_)O_4_ (0 <*x*< 1), a combination exhibiting features of both normal and inverse spinels, there is partial occupation of A and B cations across both tetrahedral and octahedral sites [55]. This structural versatility enables the incorporation of a wide range of cations, including transition metals and rare earth elements, into the A- and B-sites, enabling precise tuning of the material’s physicochemical properties. The inherent stability of the spinel lattice arises from a balanced interplay of ionic and covalent bonding, which imparts excellent thermal robustness and chemical durability. These characteristics make spinel oxides particularly suited for the harsh operational environments of SOFCs and PCFCs, including high temperatures and oxidizing or reducing atmospheres. Recent investigations reveal that deliberate modulation of cation distribution and valence states can significantly enhance proton conductivity and electrocatalytic activity, underscoring their promising role as electrode materials in PCFCs.

Electrically, spinel oxides exhibit mixed ionic–electronic conductivity, an essential attribute for high-performance air electrodes. Electronic conduction largely depends on the valence variability and electron delocalization of cations at the B-sites. Ionic conductivity, particularly for oxygen ions and protons, is intimately linked to the concentration of oxygen vacancies and proton incorporation capacity. For example, partial cation substitution can generate oxygen vacancies, which serve as conduits for oxygen ion migration via a vacancy-hopping mechanism. Proton conduction, critical for PCFC operation, is enhanced by the capacity of spinels to absorb water molecules, forming hydroxyl groups that facilitate proton transfer through hopping between adjacent oxygen ions.

Furthermore, the surface chemical properties of spinel oxides, such as specific surface area, exposed crystallographic facets, and density of catalytically active sites, substantially influence ORR kinetics. The tunability of cation composition and defect structure not only optimizes ORR activity but also improves interfacial compatibility with common electrolyte materials, including yttria-stabilized zirconia (YSZ), gadolinium-doped ceria (GDC), and yttrium-doped barium zirconate (BZY). These synergistic advantages establish spinel oxides as exceptional candidates for next-generation air electrodes in SOFCs and PCFCs, offering enhanced durability and electrochemical performance.

In the research of spinel oxides as air electrodes for SOFCs and PCFCs, density functional theory (DFT) calculations provide an indispensable approach to uncovering the intrinsic mechanisms of surface reactions at the atomic and electronic levels. By accurately evaluating the energetics and electronic structures of key elementary steps, DFT establishes causal correlations between the fundamental properties of materials and their macroscopic electrochemical performance, thereby elucidating the underlying physical origins of catalytic activity.

**Figure 4:**
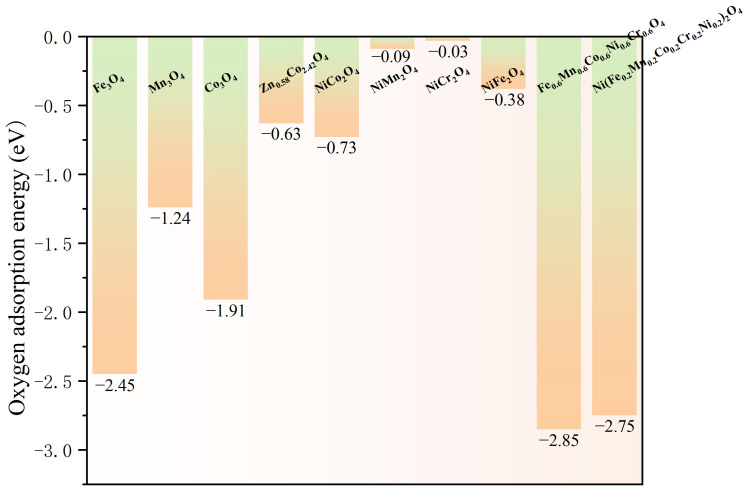
Calculated adsorption energies of O_2_ on various spinel surfaces [8,56,57].

The adsorption of oxygen molecules on the air electrode surface constitutes one of the rate-determining steps in the ORR. DFT simulations model the adsorption configurations of O_2_ on characteristic spinel surfaces and quantify the adsorption energies. Fundamentally, these calculations measure the interaction strength between the *π* antibonding orbitals of the O_2_ molecule and the electronic states of the surface active sites. Studies have demonstrated that reductions in adsorption energy closely correlate with the *d*-band center position of the surface transition metal cations. An upward shift of the *d*-band center enhances hybridization between metal *d* orbitals and O_2_ *π* orbitals, which weakens the O=O bond and facilitates adsorption. For example, the calculations on the high-entropy spinel Fe_0.6_Mn_0.6_Co_0.6_Ni_0.6_Cr_0.6_O_4_ (FMCNC) [8] reveal an O_2_ adsorption energy of −2.85 eV, markedly lower than those of its individual component oxides. This enhancement is attributed to synergistic effects arising from the high-entropy design, which induces surface electronic structure reconstruction through the coexistence of multiple elements. Similarly, Zn_0.58_Co_2.42_O_4_ (ZCO) [56] exhibits a calculated O_2_ adsorption energy of −0.63 eV, indicating thermodynamically spontaneous adsorption, whereas the traditional perovskite La_0.6_Sr_0.4_Co_0.2_Fe_0.8_O_3−δ_ (LSCF) shows a positive adsorption energy of 0.18 eV, reflecting an energy barrier. Furthermore, DFT studies demonstrate that Ni(Fe_0.2_Mn_0.2_Co_0.2_Cr_0.2_Ni_0.2_)_2_O_4_ (NFMCCN) [57] has a lower adsorption energy (−2.75 eV) and reveal that its exposed octahedral sites serve as catalytically active centers. These insights confirm that tuning the *d*-band electronic states of the surface active sites through multi-cation design is a key strategy for optimizing oxygen adsorption and activation. Figure 4 presents the O_2_ adsorption energies on various spinel surfaces.

The formation of oxygen vacancies is essential for lattice oxygen ion migration and surface oxygen exchange reactions. The vacancy formation energy is a fundamental thermodynamic parameter quantitatively reflecting the energetic barrier for lattice oxygen intercalation and deintercalation. DFT calculations provide precise values for this energy and reveal its microscopic origin. For instance, Mg^2+^ doping reduces the oxygen vacancy formation energy of Mn_1.5_Cr_1.5_O_4_ from 3.24 eV to 3.10 eV, suggesting enhanced oxygen vacancy generation propensity [58]. This decrease mainly results from local charge redistribution and lattice distortion induced by the substitution of Cr^3+^ ions with Mg^2+^ ions, which collectively weaken metal–oxygen bond strength and lower the energy cost for oxygen ion migration out of the lattice.

**Figure 5:**
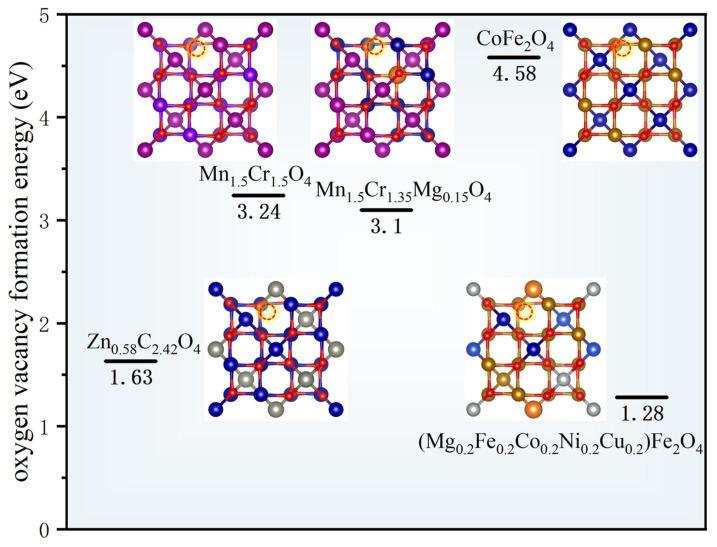
Calculated oxygen vacancy formation energies for selected spinels [56,58,59].

Additionally, DFT comparisons between the A-site high-entropy spinel (Mg_0.2_Fe_0.2_Co_0.2_Ni_0.2_Cu_0.2_)Fe_2_O_4_ (MFCNCF) and conventional CoFe_2_O_4_ (CFO) reveal pronounced differences [59]. Specifically, MFCNCF exhibits an oxygen vacancy formation energy of 1.28 eV, substantially lower than CFO’s 4.58 eV. This disparity arises from lattice distortion intrinsic to the high-entropy design. The coexistence of cations with varying valences and oxygen bonding affinities engenders non-uniform electron density distribution around oxygen atoms, inducing atomic displacements from ideal lattice positions and leading to significant structural distortion. Critically, these distortions create asymmetric and energetically unfavorable bonding environments for surface oxygen atoms, facilitating their detachment and vacancy formation. This computational insight aligns with experimental observations of increased surface-adsorbed oxygen species, providing a rigorous thermodynamic basis for the enhanced oxygen exchange kinetics and bulk oxygen diffusion performance of high-entropy spinels. Figure 5 shows oxygen vacancy formation energies for selected spinels.

In PCFC applications, the hydration capacity of air electrode materials is critical, as it determines the thermodynamic tendency for gaseous water molecules to dissociate at oxygen vacancies and generate mobile protonic defects. Hydration energy is the key metric characterizing this process. DFT calculations explicitly modeling water adsorption, dissociation, and subsequent proton incorporation into lattice oxygen sites adjacent to oxygen vacancies elucidate the underlying electronic structure mechanisms.

Research indicates that favorable hydration energy depends on two principal factors: (I) low formation energy of oxygen vacancies serving as active sites for water dissociation, and (II) the electron affinity of lattice oxygen atoms, effectively described by the O 2*p*-band center position in the electronic density of states. Notably, the O 2*p*-band center is a well-established descriptor for ORR activity in SOFC air electrodes; the closer it is to the Fermi level, the easier the removal and incorporation of oxygen atoms, which are vital steps in ORR kinetics [8,56].

DFT calculations for the novel air electrode FMCNC yield a hydration energy of −2.85 eV, significantly lower than that of the conventional spinel Mn_1.6_Cu_1.4_O_4_ (MCO) at −0.10 eV. Concurrently, the O 2*p*-band center of FMCNC is positioned at −2.12 eV, notably nearer the Fermi level compared to MCO (−2.56 eV), indicating enhanced electronic activity of lattice oxygen. This facilitates stronger interactions with H^+^ and promotes efficient proton incorporation. Similarly, DFT analysis of ZCO reports a hydration energy of −0.74 eV and an exceptionally low activation energy of 0.52 eV for the rate-determining proton migration step, much lower than the 2.94 eV reported for LSCF. Together, these DFT results provide not only quantitative energetic descriptors but also reveal that modulation of the O 2*p*-band center is the fundamental electronic structure mechanism underpinning enhanced protonation and accelerated proton migration kinetics in advanced air electrode materials.

Moreover, doping with transition metal ions represents a key strategy to tailor the local electronic structure, oxygen defect chemistry, and catalytic performance of spinels. Among the relevant structural parameters, the average metal–oxygen (M–O) bond length directly reflects bonding strength and orbital overlap between metal and oxygen atoms in the lattice. Consequently, it influences electronic conductivity and the formation energy of oxygen vacancies, as well as adsorption and activation behavior of surface oxygen species. DFT studies systematically investigate the effects of doping common transition metals (Fe, Co, Ni, Mn, Cu, Zn) in normal, inverse, and complex spinel matrices, focusing on regulation of the average M–O bond length and its intrinsic relationship to ORR activity.

**Figure 6:**
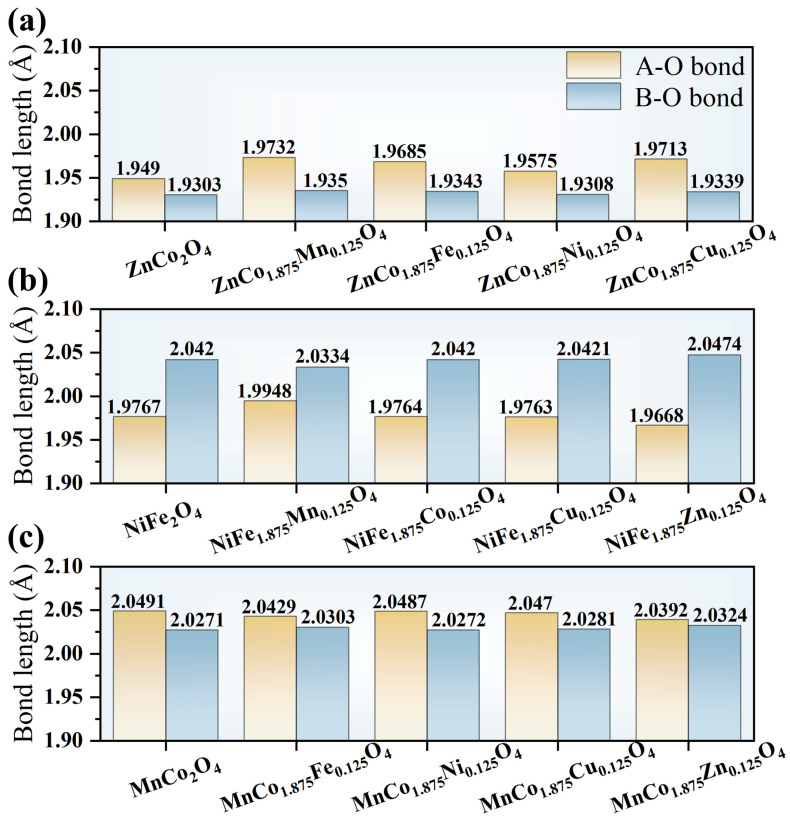
Calculated average A–O and B–O bond length of (**a**) normal spinel ZnCo_2_O_4_, (**b**) inverse spinel NiFe_2_O_4_, and (**c**) complex spinel MnCo_2_O_4_ and their transition metal-doped products.

The results demonstrate that both the type of transition metal dopant and the spinel structure significantly influence the average M–O bond length, as shown in Figure 6. Variations in bond length are closely linked to the lattice distortion and local charge redistribution induced by doping. Generally, a shortened M–O bond corresponds to enhanced metal–oxygen orbital overlap and increased covalency, which further modulates the electronic structure of the material. The effect narrows the band gap, facilitating electron transport and small polaron conduction. Moreover, reduced oxygen vacancy formation energy enhances oxygen ion migration and surface oxygen exchange kinetics. Doping also improves adsorption and activation of surface oxygen species by tuning the positions of the *d*-band center and O 2*p*-band center, optimizing oxygen molecule adsorption energies at active sites. Collectively, these effects improve ORR kinetics, leading to lower polarization resistance and higher peak power density.

## 3. Research Progress of Spinel Oxides in SOFC and PCFC Air Electrodes

### 3.1. Performance of Spinel Oxides in SOFC and PCFC Air Electrodes

The optimization of spinel oxides as air electrodes in SOFCs and PCFCs is a multi-faceted endeavor, encompassing intrinsic conductivity, thermal-mechanical compatibility, interfacial reaction kinetics, and long-term durability. Enhancing overall performance arises not from a single strategy but through the synergistic integration of compositional design, defect engineering, and microstructural regulation.

#### 3.1.1. Electrical Conductivity

Optimizing electrical conductivity hinges on promoting small polaron hopping conduction alongside establishing effective ionic transport pathways. For instance, typical spinel NiCo_2_O_4_ exhibits high electrical conductivity of approximately 36.9 S cm^−1^ at 800 °C [60]. This notable conductivity stems from the coexistence of Ni^2+^ and Ni^3+^ ions occupying octahedral sites, facilitating efficient electron hopping. More complex compositional tuning further modulates the electronic structure. High-entropy spinels such as (Mg_0.2_Fe_0.2_Co_0.2_Ni_0.2_Cu_0.2_)Fe_2_O_4_ (MFCNCF) leverage multicomponent A-site configurations to reduce the average metal–oxygen bond length and potentially narrow the electronic band gap [59]. Such modifications lower the activation energy required for small polaron hopping, thereby enhancing electrical conductivity. These examples demonstrate that controlling cation valence distributions and tailoring electronic band structures effectively enhance the intrinsic charge transport capabilities. Additional quantitative data are provided in Table 1.

#### 3.1.2. Thermal Expansion Coefficient

Spinel oxides demonstrate exceptional intrinsic thermal expansion compatibility with common electrolyte materials, underpinning their structural stability under thermal cycling. For example, the TEC of CuCo_2_O_4_ is 11.76 × 10^−6^ K^−1^, closely matching that of Sc-stabilized zirconia (SSZ) electrolyte (11.10 × 10^−6^ K^−1^) [73]. This alignment substantially mitigates interfacial stress during repeated heating and cooling cycles. Furthermore, composite strategies, such as integrating Ce_0.8_Sm_0.2_O_1.9_ (SDC) ionic conductor with FeCo_2_O_4_, enable further fine-tuning of the overall TEC, enhancing electrode–electrolyte compatibility [77]. Such composite engineering offers a practical approach to optimizing thermomechanical stability at the critical electrode/electrolyte interface. Comprehensive data are summarized in Table 2.

#### 3.1.3. Polarization Resistance

**Figure 7:**
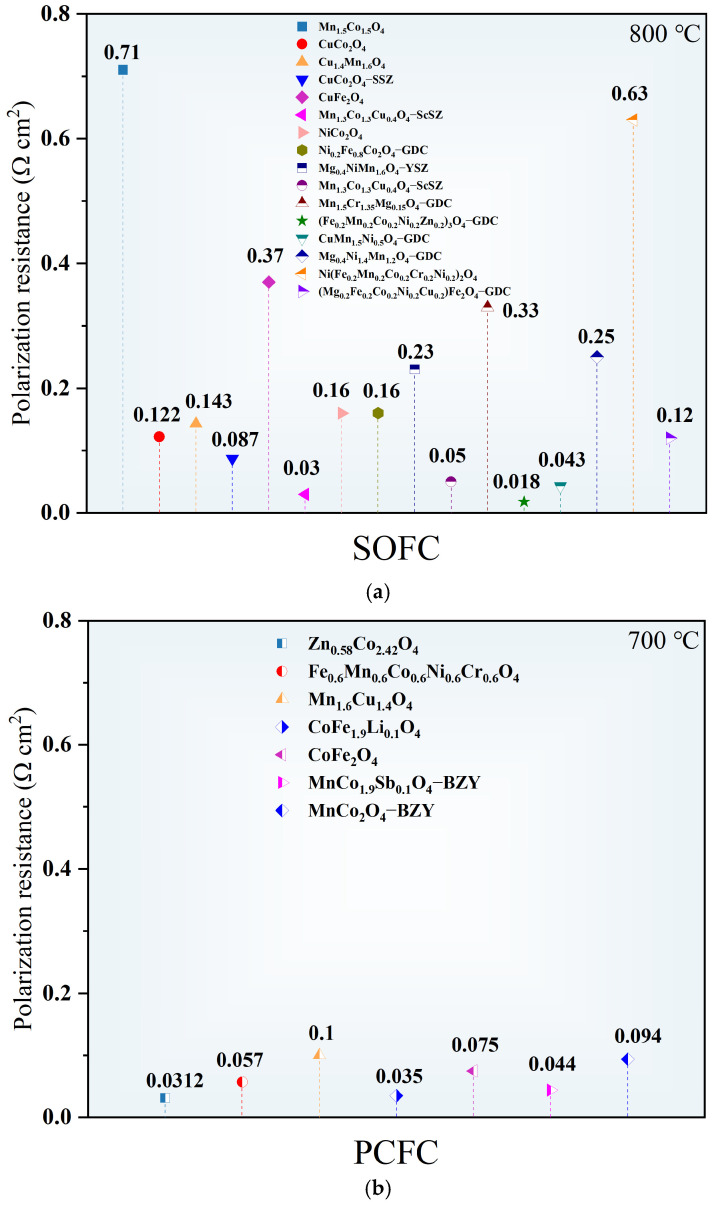
Comparison of polarization resistances of different spinel oxides as air electrodes (**a**) in SOFCs at 800 °C and (**b**) in PCFCs at 700 °C [8,51,54,56,57,58,60,61,62,63,64,67,69,70,71,73,80,81,82,83].

Reducing polarization resistance (R_p_) directly reflects accelerated ORR kinetics. This is generally achieved by increasing the density of active sites, optimizing surface chemical states, and constructing efficient ionic conduction networks. Nanostructuring is a highly effective approach to expanding electrochemical reaction interfaces. For example, in (Mn_0.6_Mg_0.4_)_0.8_Sc_0.2_Co_2_O_4_ (MMSCO), Mg and Sc doping refines the grain size to ~0.22 μm. Subsequent compositing with Pr yields MMSCO–Pr with an even smaller average grain size of 0.17 μm and a significantly increased specific surface area of 22.57 m^2^ g^−1^, thereby exposing more reactive sites for gas adsorption and electrochemical reactions [65]. In parallel, compositing with ionic conductors to establish dual ionic–electronic conduction pathways is crucial. The integration of CuMn_1.5_Ni_0.5_O_4_ with GDC greatly expands the triple-phase boundary (TPB) electrochemically active area and provides additional oxygen ion conduction channels, enabling this composite cathode to achieve an impressively low R_p_ of 0.043 Ω cm^2^ at 800 °C [70]. Figure 7 compares the polarization resistances of different spinel oxides. Furthermore, the regulation of surface chemical states is imperative. The high-entropy spinel MFCNCF exhibits a surface adsorbed oxygen to lattice oxygen ratio of 1.877, substantially exceeding that of conventional CoFe_2_O_4_ (0.929) [59], indicating a higher concentration of oxygen vacancies conducive to oxygen molecule adsorption and dissociation. Table 3 details pertinent polarization resistance data.

In fuel cells, a fundamental approach to elucidating the ORR mechanism of spinel oxide air electrodes involves systematically varying the oxygen partial pressure (*P*_*O*2_, typically ranging from 0.02 to 0.9 atm) and analyzing the corresponding electrochemical impedance spectra (EIS). By establishing a power–law relationship between polarization resistance (R_p_) and oxygen partial pressure, expressed as $R_{p}\propto R_{0}{\left(P_{O_{2}}\right)}^{-n}$, the kinetic exponent *n* serves as a critical fingerprint for identifying distinct rate-determining steps. Theoretical values of *n* correspond to specific elementary processes: *n* = 1 signifies oxygen molecule adsorption; *n* = 0.5 corresponds to dissociation of adsorbed oxygen molecules; *n* around 0.25 indicates the charge transfer step; and values near zero imply interface-controlled processes or bulk diffusion independent of oxygen partial pressure.

Empirical studies across varied material systems clearly illustrate this structure–activity relationship. For the high-entropy spinel Ni(Fe_0.2_Mn_0.2_Co_0.2_Cr_0.2_Ni_0.2_)_2_O_4_ [57], the kinetic exponents for the low and medium frequency processes are −0.48 and −0.45, respectively, indicating that the rate-determining step is the dissociative adsorption of oxygen molecules on the surface. Similarly, for another high-entropy material, (Fe_0.2_Mn_0.2_Co_0.2_Ni_0.2_Zn_0.2_)_3_O_4_ at 750 °C, and *P*_*O*2_ above 0.3 atm, the *n* value for the medium frequency process is approximately 0.32, closely matching the theoretical 0.375 associated with electron transfer to adsorbed oxygen [69]. In Ni_0.2_Fe_0.8_Co_2_O_4_ composite air electrode, as *P*_*O*2_ decreases from 1 atm to 0.21 atm, R_p_ markedly increases from 0.44 Ω cm^2^ to 1.10 Ω cm^2^, with the low frequency process exhibiting an *n* value of 0.44, indicating that migration of adsorbed oxygen to the TPB governs the kinetic limitation [67].

For copper-doped spinels, such as Mn_1.3_Co_1.3_Cu_0.4_O_4_, the *n* value is approximately 0.12, consistent with a single-electron reduction step at the TPB, signifying that charge transfer has become the principal bottleneck [64]. Likewise, in CuMn_1.5_Ni_0.5_O_4_ at 750 °C, decreasing *P*_*O*2_ from 21% to 2% results in an increase of R_p_ from 0.137 to 0.189 Ω cm^2^. Distribution relaxation time analysis reveals that the medium frequency charge transfer process accounts for over 50% of the total R_p_ at 650 °C, establishing it as the dominant rate-limiting mechanism [70]. Additional evidence arises from other systems; the air electrode modified with Mn_1.5_Co_1.5_O_4_ nanoparticles shows an *n* value of approximately 0.08 (nearly 0), indicating that the interfacial process of oxygen ions migrating from the surface into the electrolyte lattice is rate-determining [61]. For the Pr_2_CuO_4_–Pr_6_O_11_ composite air electrode at 700 °C, the kinetic exponents for high, medium, and low frequency processes are 0.11, 0.53, and 0.99, respectively, with the medium frequency oxygen dissociation step imposing the greatest resistance when *n* is approximately 0.53 [68].

In summary, the observed sequence of kinetic exponents ranging roughly from 0.5, 0.44, 0.12, to near 0 clearly demonstrates that through strategies such as high-entropy design, cation doping, and microstructural tuning, the rate-determining step of the oxygen reduction reaction on spinel air electrodes can be systematically shifted, from initial oxygen dissociation and diffusion, to migration of adsorbed oxygen, and further toward charge transfer and interfacial ion incorporation processes. This comprehensive mechanistic insight offers a solid foundation for the rational design and optimization of advanced spinel-based air electrode materials.

#### 3.1.4. Peak Power Density

**Figure 8:**
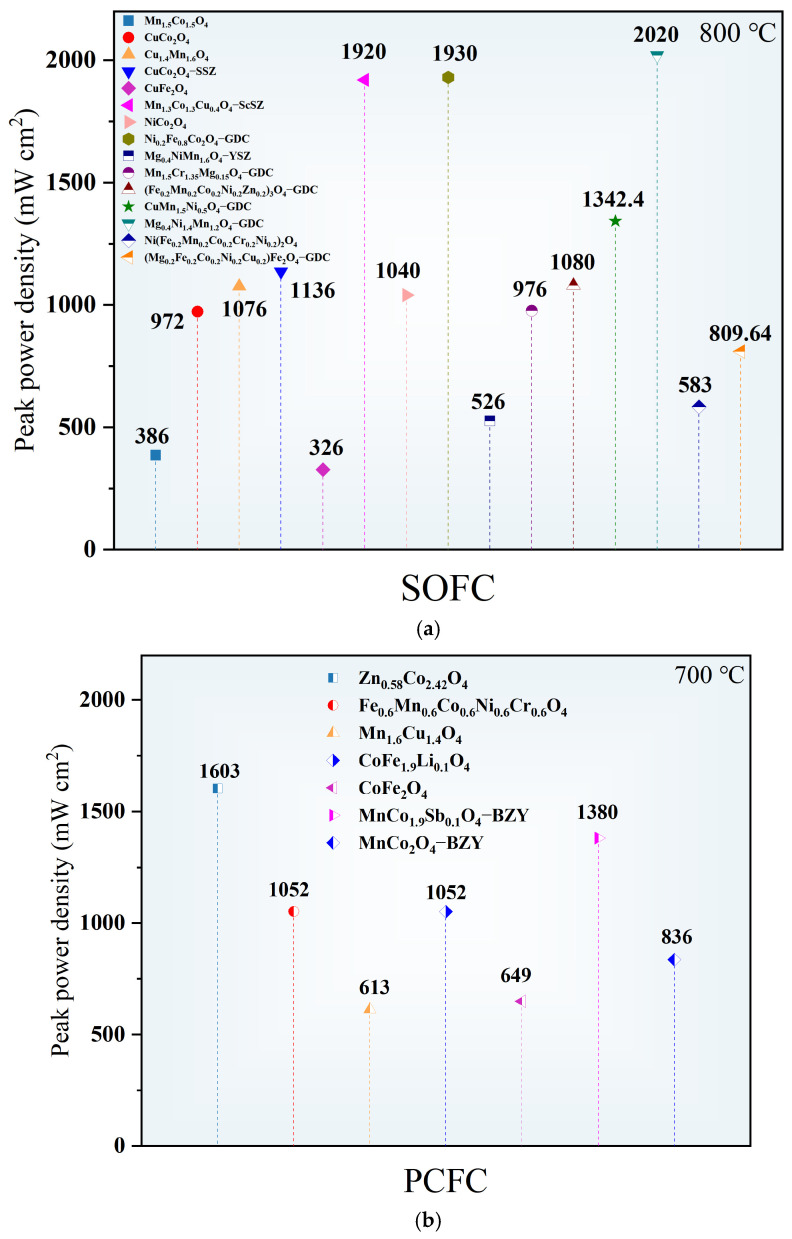
Comparison of peak power density of various spinel oxides as air electrodes (**a**) in SOFCs at 800 °C and (**b**) in PCFCs at 700 °C [8,54,56,57,58,60,61,62,63,64,67,69,70,71,73,80,81,82,83].

Peak power density (PPD) is a holistic metric of fuel cell performance, stemming from the interplay of electrical conductivity, low polarization resistance, and optimized electrode microstructure. Intrinsic catalytic activity and proton/oxygen ion conduction form the foundation, while cathode/electrolyte interface optimization critically reduces transport resistance. For instance, a single cell employing the advanced MMSCO–Pr cathode achieved a PPD of 1144.1 mW cm^−2^ at 750 °C, attributable to its exceptionally low area-specific resistance (ASR) of 0.11 Ω cm^2^, alongside finely tuned nanostructure [65]. Similarly, Zn_0.58_Co_2.42_O_4_ (ZCO) demonstrates significant interface-optimization-driven performance improvements [56]. Its relatively low melting point (~913 °C) facilitates enhanced cathode–electrolyte contact during co-firing at 1000 °C, markedly lowering interfacial ohmic resistance (R_ohm_ decreased from 0.165 Ω cm^2^ sintered at 900 °C to 0.104 Ω cm^2^ sintered at 1000 °C). The optimized interface, combined with the inherent superior oxygen reduction and proton conduction properties of ZCO, enhanced PPD for single cells from 1295 to 1603 mW cm^−2^. Figure 8 compares the peak power density of various spinel oxides. These cases illustrate that substantial improvements in PPD arise not only from intrinsic material activity but also critically from minimizing interfacial resistance via adept microstructural and processing optimizations. Additional PPD values are compiled in Table 4.

#### 3.1.5. Long-Term Stability

Long-term operational stability relies on inherent chemical stability, sustained structural integrity, and robust electrode–electrolyte adhesion. Spinel materials such as ZCO and CoFe_1.9_Li_0.1_O_4_ have exhibited no formation of secondary phases after rigorous exposure to harsh atmospheres, such as 10% CO_2_ or 30% H_2_O at 600 °C for periods ranging from 10 to 50 h, indicating their excellent environmental tolerance [56,81]. Operationally, NiCo_2_O_4_ cathodes maintained stable performance over 45 h at 700 °C under a current density of 150 mA cm^−2^ without noticeable degradation [60]. The structural stability benefit imparted by high-entropy designs is notable; nanostructured high-entropy spinel MFCNCF showed negligible performance degradation or particle coarsening following 240 h of operation at 750 °C [83,84,85,86,87,88,89]. This resilience is attributed to high configurational entropy, which effectively suppresses element segregation and grain growth at elevated temperatures. Lastly, optimized thermal expansion matching, as detailed previously, preserves interfacial integrity during thermal cycling and mechanically prevents delamination, collectively ensuring long-term durability.

### 3.2. Chemical Design of Spinel Oxides in SOFC and PCFC Air Electrodes

From a material design standpoint, spinel oxides offer considerable flexibility for doping at both A- and B-sites. Substituent cations can enhance phase stability, electrical conductivity, or proton uptake; modulate the thermal expansion coefficient; and introduce oxygen vacancies or electronic defects within the lattice. The following sections elaborate on doping strategies at each site.

#### 3.2.1. A-Site Doping

Tetrahedral A-site doping constitutes a vital approach for tuning the properties of spinel oxides, underpinned by a common physicochemical mechanism. This mechanism involves introducing heterovalent cations with specific ionic sizes, oxidation states, and electronic configurations (especially *d*-electron occupancy) into the tetrahedrally coordinated sites. Such doping perturbs the local coordination environment of the A-site, altering bonding characteristics such as bond length, strength, and covalency of the A–O bond. This primary perturbation cascades into a series of secondary effects, including structural stabilization or reconstruction (e.g., suppression of Jahn–Teller distortions), rearrangement of B-site cation valences and distributions driven by charge compensation or crystal field stabilization energy (CFSE), and modulation of the electronic band structure and polaron hopping pathways. Collectively, these phenomena synergistically optimize critical material properties, reducing energy barriers for proton or oxygen ion migration, enhancing electronic conductivity via small polaron hopping, and improving surface oxygen exchange reaction kinetics. While this theoretical framework applies broadly, specific tetrahedral doping strategies vary according to target performance metrics and operational pathways.

**Figure 9:**
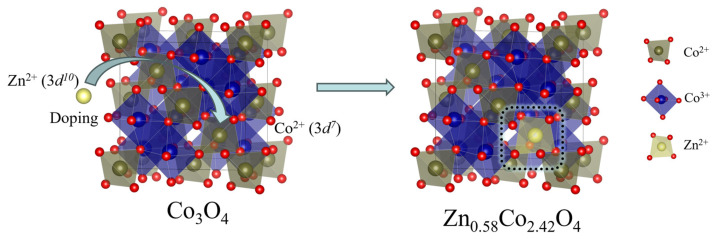
Schematic diagrams of Zn^2+^ doped Co_3_O_4_ form Zn_0.58_Co_2.42_O_4_.

Strategies directly enhancing proton conduction and surface reactivity focus on creating local chemical environments conducive to proton (H^+^) transport. For example, in Zn_0.58_Co_2.42_O_4_ [49], substituting Jahn–Teller active Co^2+^ (3*d*^7^) at tetrahedral sites with Jahn–Teller inactive Zn^2+^ (3*d*^10^) stabilizes the crystal structure by reducing distortions (Figure 9). Concurrently, Zn^2+^’s lower charge density weakens lattice oxygen bonding, substantially lowering the energy barriers for proton rotation and hopping on adjacent oxygen ions (0.52 and 0.12 eV, respectively).

Conversely, conventional A-site doping primarily exploits the size mismatch and charge differences between dopant and ions to finely tune lattice parameters, electronic structure, and oxygen defect chemistry. A representative example is (Mn_0.6_Mg_0.4_)_0.8_Sc_0.2_Co_2_O_4_, where Mg^2+^ partial substitution for Mn mitigates Jahn–Teller distortion and stabilizes the spinel framework; simultaneously, the incorporation of higher-valent Sc^3+^ introduces cation vacancies or drives oxidation of Co/Mn ions to higher valence states for charge compensation, significantly reducing the formation energy of oxygen vacancies [65]. The resulting synergy between structural stabilization and charge perturbation primarily optimizes bulk oxygen ion migration networks and thermal expansion compatibility.

Another category of strategies targets electronic structure modulation, band characteristics, and intrinsic charge transport by altering A–O bond covalency to affect polaron hopping and electron mobility. For instance, Ni_0.4_Zn_0.6_Fe_2_O_4_ [90] achieves improved electronic conductivity via partial substitution of tetrahedral Ni^2+^ with Zn^2+^ (*d*^10^ closed-shell), which alters electron density distribution, weakens A–O covalency, and influences B-site Fe^3+^ electronic states through crystal field effects. Similarly, in Cu_0.5_MnCo_1.5_O_4_ [61], Cu^2+^ at A-sites acts as a charge compensator, elevating B-site Mn oxidation states towards Mn^4+^(*t*_2_*g*^3^*eg*^0^), which enhances electron acceptance and catalytic activation of oxygen molecules. This process effectively accelerates oxygen reduction reactions by tuning B-site catalytic centers.

Some strategies focus explicitly on reducing electronic conductivity distances through geometric lattice adjustments. For example, Mg^2+^ doping in Mn_1.5_Cr_1.5_O_4_ [58] preferentially occupies tetrahedral sites due to its relatively small ionic radius (0.72 Å), resulting in lattice contraction and decreased Mn–O bond lengths. This contraction lowers activation barriers for small polaron hopping between Mn^3+^/Mn^2+^ sites, significantly improving intrinsic electronic conductivity.

**Figure 10:**
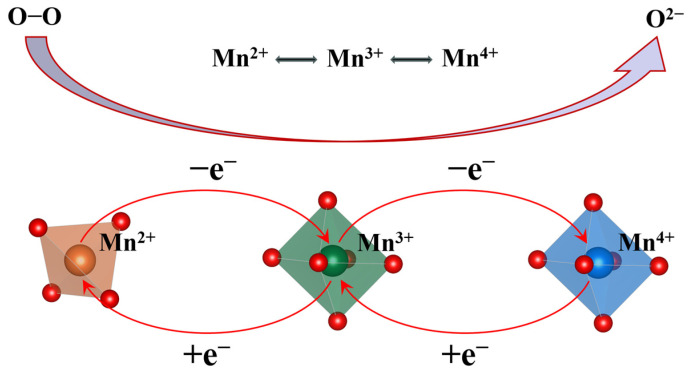
Coordination conversion of Mn in Mg_*x*_NiMn_2−*x*_O_4_.

The most systematic understanding of these phenomena derives from crystal field theory, highlighting A-site doping as a thermodynamic trigger for cation redistribution and valence states re-equilibration throughout the spinel lattice. The Mg*_x_*NiMn_2−*x*_O_4_ system exemplifies this mechanism [54]: During high-temperature synthesis, Mg^2+^ (*d*^0^, negligible CFSE preference) occupies tetrahedral A-sites, displacing Ni^2+^ (*d*^8^, strong octahedral preference due to higher CFSE) to octahedral B-sites. Concurrently, charge neutrality demands rearrangement of Mn valences at the B-site, wherein Jahn–Teller active Mn^3+^ partially converts to Mn^2+^ or Mn^4+^ species (Figure 10). This process generates abundant multivalent Mn^2+^/Mn^3+^/Mn^4+^ pairs that facilitate small polaron conduction and promote the formation of oxygen vacancies, significantly enhancing both electronic and oxygen ion transport capabilities. Thus, A-site doping catalyzes comprehensive intrinsic property re-engineering within the spinel oxide.

#### 3.2.2. B-Site Doping

Octahedral-site (B-site) doping plays a pivotal role in the functional design of spinel oxides. Unlike A-site doping, which mainly induces indirect effects, B-site doping directly modifies the octahedral sites as the principal active center for oxygen reduction reaction (ORR). This direct modification influences the material’s oxygen defect chemistry, intrinsic charge transport properties, and surface catalytic activity. Specifically, B-site dopants alter metal–oxygen (M–O) bond strength, local electron density, and the crystal field environment, thereby enabling precise tuning of essential material characteristics.

While all B-site doping strategies share this common mechanistic basis, they can be categorized into distinct groups according to the dopant ions’ properties and the resulting cascade of effects on the host lattice.

One major strategy aims at directly regulating oxygen vacancy concentration and enhancing bulk ion migration pathways. This is typically achieved by introducing dopants with ionic size mismatch or lower valence states, which induce lattice strain and drive charge compensation mechanisms to generate and activate ion transport channels. For instance, in CoFe_1.9_Li_0.1_O_4_ [81], the incorporation of low-valency Li^+^ into octahedral sites mandates charge neutrality preservation, triggering oxidation of adjacent Fe^3+^ and Co^2+^ ions to higher valence states. This process not only increases redox activity but also creates positively charged oxygen vacancies, thereby substantially raising the surface-adsorbed oxygen species ratio from 0.56 to 0.96. Consequently, ORR kinetics are markedly accelerated, and polarization resistance sharply decreases to 0.035 Ω cm^2^. Similarly, CoGd_0.2_Fe_1.8_O_4_ [91] exploits the larger ionic radius of Gd^3+^ substituting for Fe^3+^, causing lattice expansion and local strain, which weakens M–O bonds and lowers oxygen vacancy formation energy, facilitating long-range oxygen ion migration. In MnCo_1.9_Sb_0.1_O_4_ [82], Sb^3+^ doping similarly induces lattice expansion that broadens proton migration channels, leading to respective enhancements of ~64% in proton diffusion coefficient and ~73% in surface exchange rates.

**Figure 11:**
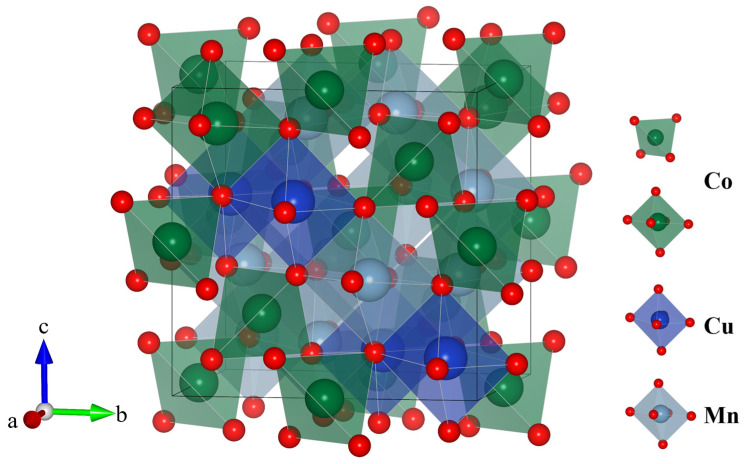
Crystal structure of MCCO spinel.

**Figure 12:**
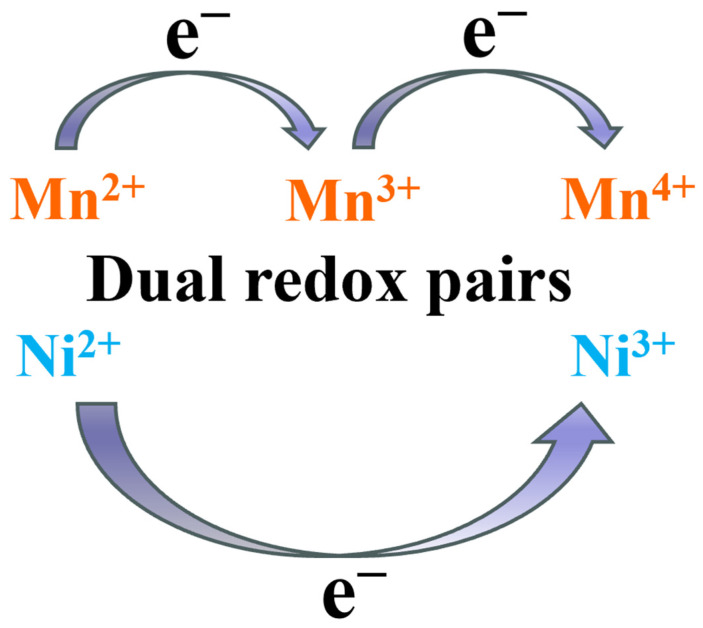
Dual redox pairs of Mg_0.4_Ni_*x*_Mn_2.6–*x*_O_4+δ_.

Complementing ion transport optimization, another advanced approach centers on reconstructing the material’s electronic conduction network via introduction of multivalent dopants. These dopants establish synergistic, multi-component redox couples with host ions, fundamentally innovating the charge transport mechanism. For instance, in Mn_1.3_Co_1.3_Cu_0.4_O_4_ [64], Cu doping drives Mn ions entirely into octahedral sites and stabilizes them at higher valence states. This also forms a synergistic mixed-valence system comprising Cu^+^/Cu^2+^ and Mn^3+^/Mn^4+^ couples (Figure 11), effectively narrowing the band gap from 3.8 eV to 3.2 eV. This electronic structure modification elevates conductivity to 143 S cm^−1^ at 700 °C, while yielding an exceptionally low polarization resistance of 0.03 Ω cm^2^. Similarly, CuMn_1.5_Ni_0.5_O_4_ [70] incorporates Ni^2+^ ions that generate dual redox couples (Cu^+^/Cu^2+^ and Mn^3+^/Mn^4+^) through charge compensation, enabling multidimensional electron hopping pathways. In the Mg_0.4_Ni_*x*_Mn_2.6−*x*_O_4+δ_ [71], both Ni^2+^/Ni^3+^ redox pairs and manganese valence network act as dual active centers (Figure 12), dynamically managing oxygen vacancies via charge self-compensation and optimizing the balance between electronic conductivity (reaching 68 S cm^−1^ at 800 °C) and ionic transport.

Beyond transport properties, maintaining sustained high performance requires long-term structural stability. This challenge is addressed by a third category of strategies that exploit the specific electronic configurations of dopant ions to stabilize the lattice, a concept known as crystal field engineering. For instance, theoretical studies on Co-doped NiFe_2_O_4_ [92] reveal that low-spin Co^3+^ (*t*_2_*g*^6^*eg*^0^) preferentially occupies octahedral sites, yielding an energetically favorable lattice configuration. The incorporation of Co^3+^ not only reinforces structural stability but also suppresses deleterious lattice distortions and degradation induced by Jahn–Teller active ions, such as Mn^3+^ and Ni^3+^, by modulating their concentration and electronic states. In spinel systems containing Jahn–Teller active ions such as Mn^3+^, high-entropy or multi-element doping strategies can effectively mitigate detrimental structural distortions, thereby enhancing structural stability. This stabilization primarily stems from two aspects: first, the lattice strain and chemical disorder induced by high configurational entropy; for instance, in high-entropy spinels, the random distribution of multiple cations at the A-site creates a disordered local stress field, which counteracts the long-range ordered Jahn–Teller distortion that is typically driven by a single Jahn-Teller-active ion [83]. Second is the modulation of ionic valence states and coordination environments through deliberate doping. In MnCo_1.9_Sb_0.1_O_4_, the incorporation of high-valent Sb^5+^ can regulate the oxidation state of Mn, potentially reducing the concentration of Mn^3+^ and thus reducing the instigation of the Jahn–Teller effect [82]. This combined enhancement in chemical and mechanical robustness under harsh fuel cell operating conditions establishes a solid foundation for long-term electrode stability from both electronic and crystal field engineering perspectives.

#### 3.2.3. High-Entropy Engineering

High-entropy engineering marks a substantial advancement in the design strategy for spinel materials. Unlike traditional single-element doping, which focuses on localized modifications, this approach explores the collective effects arising from the incorporation of five or more principal elements in near-equimolar ratios within a single crystal lattice. Current research attributes the underlying mechanisms of high-entropy spinels primarily to the interplay of the following interrelated aspects: (I) Entropy stability effect [87]: The substantial configurational entropy contribution effectively reduces the Gibbs free energy of the system, thermodynamically favoring the formation and stabilization of a single solid-solution phase while suppressing element segregation and secondary phase precipitation at elevated temperatures; (II) Lattice distortion effect [93]: The random distribution of atoms with diverse ionic radii and chemical properties generates significant local stress fields and lattice strain. Such distortions influence the diffusion kinetics of bulk ions, often resulting in modified transport behaviors; (III) Synergistic electronic structure modulation [94]: Interactions among *d*-orbital electrons of multiple transition metals fine-tune the overall electronic structure, impacting factors such as the position of the O 2*p* band center and metal–oxygen bond covalency. These alterations can enhance electrical conductivity and catalytic activity.

The synergy among these effects underpins the capability of high-entropy spinels to simultaneously improve multiple performance metrics, including catalytic activity and structural stability. Existing literature reveals several strategic pathways within high-entropy engineering, each emphasizing specific elemental combinations and microstructural design principles.

**Figure 13:**
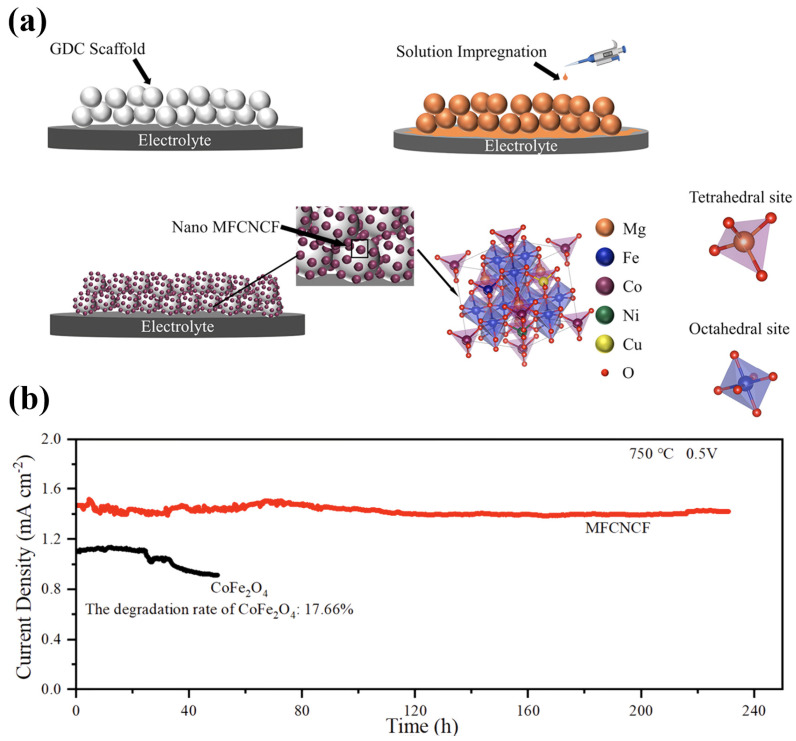
(**a**) Schematic diagram of the impregnation process for preparing nanostructured MFCNCF; (**b**) Long-term stability test of i–30CoFe_2_O_4_–GDC and i–30MFCNCF–GDC single cells under a constant voltage of 0.5 V at 750 °C. Source: Reprinted from [83], with permission from American Chemical Society (Copyright 2025, American Chemical Society).

A prominent research direction investigates the potential of high-entropy compositions to stabilize nanostructures. This exploits the sluggish bulk diffusion kinetics inherent to multi-principal element solid solutions, effectively inhibiting nanoparticle coarsening at elevated temperatures. For example, nano-sized (Mg_0.2_Fe_0.2_Co_0.2_Ni_0.2_Cu_0.2_)Fe_2_O_4_ high-entropy particles deposited onto a GDC scaffold (Figure 13a) [83] demonstrate that the heterogeneous occupancy of multiple elements at the A-site markedly reduces metal ion diffusion rates. Consequently, the nanoparticles maintain small sizes and high dispersion even after 240 h of operation at 750 °C (Figure 13b). This approach addresses a critical challenge in sustaining long-term thermal stability of nanostructured electrode materials.

**Figure 14:**
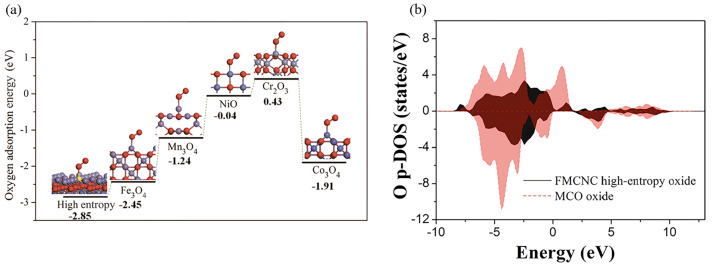
(**a**) Adsorption energy of O_2_ on the surface of FMCNC high-entropy oxide as well as on the Fe_3_O_4_, Mn_3_O_4_, Co_3_O_4_, NiO, and Cr_2_O_3_ surfaces; (**b**) O p-DOS for FMCNC high-entropy oxide and MCO oxide. Source: Reprinted from [8], with permission from Springer Nature (Copyright 2022, Springer Nature).

Another focus area centers on enhancing intrinsic electrocatalytic performance via multi-element synergy. This strategy typically incorporates multiple transition metals at catalytically active sites, especially B-sites, to leverage the “cocktail effect” in optimizing surface reaction dynamics. For instance, theoretical calculations for Fe_0.6_Mn_0.6_Co_0.6_Ni_0.6_Cr_0.6_O_4_ indicate the O 2*p* band center situated closer to the Fermi level and a calculated O_2_ adsorption energy of −2.85 eV, surpassing that of single-component oxides. Such findings suggest that multi-element synergy improves oxygen reduction activity (Figure 14) [8]. Similarly, Ni(Fe_0.2_Mn_0.2_Co_0.2_Cr_0.2_Ni_0.2_)_2_O_4_ [57] exhibits a high surface adsorbed oxygen to lattice oxygen ratio (O_ads_/O_lat_ = 1.42) as revealed by X-ray photoelectron spectroscopy (XPS), while density functional theory (DFT) calculations confirm strong oxygen adsorption energy (−2.75 eV). Together, these results underscore the ability of high-entropy designs to enhance adsorption and activation processes for oxygen species through a complex landscape of surface active sites.

Finally, several studies demonstrate that high-entropy spinels achieve significant overall performance improvements by concurrently attaining high catalytic activity, rapid charge transport, and excellent structural stability via judicious compositional design. For instance, (Fe_0.2_Mn_0.2_Co_0.2_Ni_0.2_Zn_0.2_)_3_O_4_ exhibits outstanding features, including a high surface oxygen activity (O_ads_/O_lat_ = 1.61), low polarization resistance (0.018 Ω cm^2^ at 800 °C), and excellent structural stability over prolonged operation [69]. These outcomes suggest that the high-entropy strategy effectively mitigates the typical performance trade-offs of conventional materials, offering a promising compositional design space for developing advanced electrode materials.

## 4. Design Principles and Application Prospects of High-Entropy Spinel Oxides

High-entropy engineering, as an emerging material design paradigm, aims to stabilize simple solid-solution structures by harnessing high configurational entropy through the incorporation of five or more principal elements in near-equimolar ratios [95]. This concept spans a variety of material classes, including high-entropy alloys, oxides, ceramics, and more. Among these, high-entropy spinel oxides have attracted considerable attention as promising candidates for air electrodes in SOFC PCFCs [96,97,98,99,100,101].

High-entropy alloys serve as the foundational category of high-entropy materials. Defined as alloys containing five or more principal elements, each in atomic percentage ranging from 5% to 35%, high-entropy alloys leverage configurational entropy to stabilize solid-solution phases that are otherwise difficult to form in conventional alloys. This principle laid the groundwork for subsequent research into high-entropy oxide. The configurational entropy can be quantified by the equation:

(1) $$\Delta S_{mix}=-R\sum_{i=1}^{n}x_{i}lnx_{i}$$

where n denotes the number of constituent elements, R is gas constant, and $x_{i}$ represents the mole fraction of ith element. Research on high-entropy oxides commenced in 2015 when Rost et al. [93] successfully synthesized single-phase rock salt structured high-entropy oxides (Co, Cu, Mg, Ni, Zn)O via high-temperature solid-state reactions. Subsequent studies have developed diverse synthesis routes, such as coprecipitation and the sol–gel method, and expanded the crystal structures scope to include fluorite, perovskite, and spinel phases. Among these, high-entropy spinel oxides have emerged as a focal point for SOFC and PCFC air electrode materials, owing to their distinctive structural and functional advantages.

**Figure 15:**
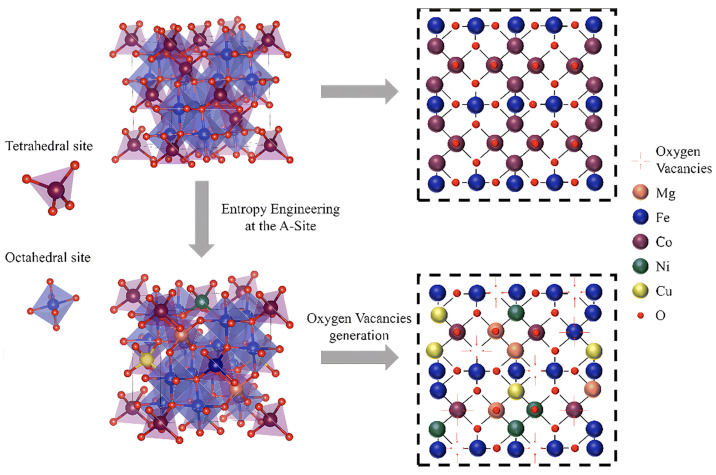
A schematic diagram of the lattice distortion in O-neighbored octahedral and tetrahedral sublattices resulting in oxygen vacancy formation of CoFe_2_O_4_ and MFCNCF. Source: Reprinted from [59], with permission from Royal Society of Chemistry (Copyright 2024, Royal Society of Chemistry).

Core investigations into high-entropy spinel oxides have focused on their structural attributes and underlying mechanisms of action. Dąbrowa et al. [102] first synthesized single-phase high-entropy spinel (Co, Cr, Fe, Mn, Ni)_3_O_4_, confirming its structural homogeneity via X-ray diffraction and related techniques. The remarkable thermal stability stems from the nearly equivalent bond strengths of A–O and B–O bonds, which confer excellent chemical compatibility and ensure long-term operational durability in SOFCs and PCFCs. From a mechanistic perspective, the disordered occupancy of multivalent transition metal cations across octahedral and tetrahedral sites causes electron cloud redistributions around oxygen atoms. This lattice distortion fosters the formation of oxygen vacancies, which serve as critical conduction channels for oxygen ions. Figure 15 schematically illustrates the lattice distortion alongside entropy-driven oxygen vacancy generation in spinel oxides. Additionally, the high-entropy effect’s characteristic sluggish atomic diffusion suppresses particle agglomeration and phase separation of air electrode materials at elevated temperatures [59,84,85,86,87,88,89,103,104], thereby further bolstering material stability.

The exceptional stability of high-entropy spinel oxides as air electrodes in SOFCs and PCFCs arises primarily from their thermodynamic stabilization driven by high configurational entropy. A material is classified as high-entropy when its configurational entropy change, $\Delta S_{mix}$, exceeds 1.5 R. According to the Gibbs free energy equation, G=H−TS, a substantial increase in configurational entropy elevates the total entropy S, making the −TS term the dominant factor that lowers the Gibbs free energy. This entropic contribution is sufficiently large to offset the enthalpic penalty H associated with lattice strain and chemical mismatch among multiple dissimilar cations, thereby rendering the single-phase solid solution thermodynamically more stable than its phase-separated counterpart. This phenomenon is commonly termed the entropy stabilization effect.

The effect manifests first in the suppression of phase separation during synthesis, stabilizing the single-phase structure. Experimental evidence demonstrates that entropy engineering at the A-site enables the high-entropy composition (Mg_0.2_Fe_0.2_Co_0.2_Ni_0.2_Cu_0.2_)Fe_2_O_4_ (MFCNCF) to form a pure spinel phase, whereas the low-entropy analog (Mg_0.25_Co_0.25_Ni_0.25_Cu_0.25_)Fe_2_O_4_ (MCNCF) contains Fe_2_O_3_ impurity phase. This contrast directly confirms that elevated configurational entropy effectively expands the solid-solution limit and inhibits cation segregation [59]. Similarly, investigations of the (Co, Cr, Fe, Mn, Ni)_3_O_4_ system reveal that a single spinel phase is readily synthesized via high-temperature solid-state reaction. In situ X-ray diffraction confirms no phase transformations from room temperature to 1273 K, highlighting the intrinsic, wide-temperature-range phase stability imparted by high-entropy design [105].

Second, temperature amplifies the entropy stabilization effect. In the Gibbs free energy equation, temperature *T* multiplies the entropy term, thus increasing *T*, which proportionally enhances the contribution of configurational entropy to thermodynamic stability. This principle not only highlights the advantage of high-temperature synthesis for obtaining stable single-phase solid solutions but also explains why the medium to high operational temperature range of SOFCs and PCFCs (650–800 °C) is ideal for fully leveraging the entropy stabilization effect to maintain long-term microstructural integrity.

Under these operational conditions, entropy stabilization primarily manifests as a pronounced suppression of microstructural degradation. The random multi-element occupancy of lattice sites induces lattice distortion and heterogeneous, disordered stress fields, significantly raising the activation energy barrier for atomic diffusion and slowing elemental migration. This kinetic stabilization is corroborated by durability tests: for instance, a nanostructured MFCNCF air electrode prepared by impregnation retained its polarization resistance and power density without measurable degradation after 240 h at 800 °C, fully preserving its original nanostructure. In contrast, a conventional CoFe_2_O_4_ nanoparticle electrode suffers over 17% performance loss within 50 h under identical conditions, caused by particle agglomeration and coarsening [83]. Likewise, the high-entropy spinel Fe_0.6_Mn_0.6_Co_0.6_Ni_0.6_Cr_0.6_O_4_ (FMCNC) air electrode exhibits exceptional operational stability during fuel cell tests [8]. Collectively, these findings establish entropy stabilization as a broadly effective strategy to enhance the high-temperature aging resistance of nanostructured electrodes.

It is also notable that certain specific multi-element combinations among high-entropy spinel electrodes not only confer structural stability but also appear thermodynamically favorable for oxygen adsorption. For instance, the five-element transition metal systems containing Fe, Mn, Co, Ni, and Cr has demonstrated outstanding performance across several studies [8,57,59,83]. DFT calculations provide preliminary evidence supporting the advantages of these combinations. Reported O_2_ adsorption energies for FMCNC (−2.85 eV) and Ni(Fe_0.2_Mn_0.2_Co_0.2_Cr_0.2_Ni_0.2_)_2_O_4_ (NFMCCN) (−2.75 eV) are both lower than those of their respective binary or single oxides, indicating enhanced oxygen activation energetics. Another study reports that the A-site high-entropy spinel MFCNCF has a substantially lower oxygen vacancy formation energy (1.28 eV) compared to its low-entropy analog CoFe_2_O_4_ (4.58 eV), suggesting its surface more readily forms active oxygen vacancies for catalysis.

Further analysis attributes these favorable thermodynamic properties to the high configurational entropy and resultant modifications to the local bonding environment. Theoretically, the incorporation of multiple elements increases the configurational entropy $\Delta S_{mix}$, stabilizing solid solutions and suppressing cation segregation, as confirmed by the formation of single-phase spinel structures in high-entropy samples like MFCNCF and FMCNC. Detailed structural characterization reveals that multi-component design alters the local bond environment, with the average metal–oxygen (M–O) bond length as an especially important parameter. For instance, MFCNCF exhibits an average metal–oxygen bond length of 2.048 Å, slightly shorter than that of its reference sample CoFe_2_O_4_ (2.050 Å). Similarly, in the B-site high-entropy system NFMCCN, the average M–O bond length is 2.004 Å, shorter than those of its binary references NiFe_2_O_4_ (2.028 Å) and NiMn_2_O_4_ (2.046 Å). This bond shortening enhances the covalency of metal–oxygen and promotes charge transfer. Meanwhile, the presence of multiple multivalent transition metals establishes continuous redox couples, accelerating electron transfer during the oxygen reduction reaction [106,107]. Furthermore, the random occupation of lattice sites by ions with significantly different radii generates substantial lattice distortion and localized stress fields. This distortion reduces the formation energy of oxygen vacancies and thereby greatly increases the surface oxygen vacancy concentration; XPS surface analysis confirms that high-entropy materials have a higher proportion of adsorbed oxygen species; the ratio of adsorbed oxygen to lattice oxygen is 1.877 in MFCNCF compared to 0.929 in CoFe_2_O_4_. These combined structural and electronic modifications synergistically enhance oxygen adsorption and activation processes, underpinning the superior catalytic performance of high-entropy spinel air electrodes.

Despite these advances, challenges remain in high-entropy spinel oxide research. Conventional synthesis methods often yield powders with broad and uneven particle size distributions. Moreover, the oxygen ion conductivity of spinel oxides typically lags behind that of perovskite counterparts, motivating ongoing efforts to improve performance through combined entropy and defect engineering strategies. Furthermore, the precise structure–property relationship governing high-entropy materials within SOFC and PCFC systems remains incompletely understood, and the exploration of high-entropy ceramic air electrodes in PCFCs is still in its nascent stages. Overall, high-entropy spinel oxides offer promising prospects in the SOFC and PCFC domains due to their unique structural and functional advantages, but future work must deepen mechanistic insights and optimize synthesis processes.

Although significant progress has been made in tuning spinel oxide structures and enhancing their properties, notable challenges persist for their practical application as air electrode materials in SOFCs and PCFCs. First, spinel particles tend to undergo aggregation or sintering during high-temperature fabrication or prolonged operation, which diminishes specific surface area and active site exposure, undermining nanostructure advantages and degrading catalytic kinetics over time. Second, the high density of grain boundaries characteristic of nanostructured spinels can introduce additional ion transport barriers: in SOFCs, impeding oxygen ion migration; in PCFCs, obstructing proton conduction at the electrode–electrolyte interface, thus increasing overall cell impedance. Third, interface compatibility between spinel electrodes and electrolytes (e.g., YSZ for SOFCs, BZCY for PCFCs) remains a critical bottleneck. The large specific surface area of nanostructures exacerbates interfacial chemical reactions that often lead to the formation of low-conductivity secondary phases (such as spinel-zirconia solid solutions) and induce interfacial stresses, compromising electrode structural integrity. Finally, achieving controlled synthesis of spinels with uniform particle size, excellent dispersion, and robust mechanical strength at scale remains technically challenging.

## 5. Conclusions

This review presents a comprehensive overview of recent research advances in spinel-type oxides as air electrode materials for SOFCs and PCFCs. Capitalizing on their intrinsic structural flexibility and adjustable defect chemistry, spinel oxides exhibit significant promise in optimizing electrical conductivity, improving thermal-mechanical compatibility, and enhancing ORR activity. Notably, emerging strategies such as high-entropy engineering provide innovative avenues to achieving electrodes that deliver both high catalytic activity and outstanding long-term stability.

Nevertheless, several critical challenges remain. First, the microscopic mechanisms governing proton conduction and surface reactions in spinel materials, especially in PCFC environments, are not yet fully understood. Strengthening theoretical frameworks through advanced computational simulations and experimental investigations is imperative. Second, long-term chemical compatibility and interfacial stability with electrolytes such as YSZ and BZCY continue to limit performance and durability, necessitating systematic studies and novel interface engineering approaches. Third, scalable synthesis of spinel oxides featuring uniform morphology, tailored porosity, and robust mechanical properties remains an important practical hurdle for technology commercialization.

Looking forward, future research should focus on:

(i) advanced characterization and modeling: Integrate in situ/operando spectroscopic and microscopic techniques with DFT calculations to elucidate atomic-scale charge transport and reaction mechanisms.
(ii) interface and composite engineering: Develop innovative composite architectures and defect-engineered interfaces to synergistically enhance electrochemical performance and durability.
(iii) cross-scale investigations: Correlate microstructural evolution with macroscopic electrochemical degradation to inform rational material design strategies.

In summary, spinel oxides represent a distinctive and versatile class of functional materials. Continued exploration of their fundamental structure–property relationships will not only advance scientific understanding but also accelerate the development of next-generation, high-performance ceramic fuel cells.

## Figures and Tables

**Table 1:** The electrical conductivity of different spinel oxides as reported in the literature.

Components			σ (S cm^−1^)					Year	Reference
Air Electrode	Electrolyte	Fuel Electrode	800 °C	750 °C	700 °C	650 °C	600 °C		
Mn_2_CoO_4_	YSZ ^1^	Ni–YSZ	21	/	/	/	/	2013	[61]
Mn_1.5_Co_1.5_O_4_	YSZ	Ni–YSZ	22.3	/	/	/	/		
MnCo_2_O_4_	YSZ	Ni–YSZ	71.8	/	38.6	/	/		
Cu_1.4_Mn_1.6_O_4_	SSZ ^2^	Ni–SSZ	78	75	72	67	60	2016	[62]
CoFe_2_O_4_	LSGM ^3^	Ni–SDC ^4^	0.49	0.26	0.2	0.13	0.1	2019	[63]
NiFe_2_O_4_	LSGM	Ni–SDC	0.09	0.07	0.06	0.05	0.05		
CuFe_2_O_4_	LSGM	Ni–SDC	2.2	1.6	1.2	0.75	0.49		
Mn_1.3_Co_1.3_Cu_0.4_O_4_–ScSZ	ScSZ ^5^	Ni–YSZ	168	143	122	103	8.4	2019	[64]
MnCo_2_O_4_	YSZ	Ni–YSZ	80.9	66.7	49.8	35.1	23.1	2021	[65]
(Mn_0.6_Mg_0.4_)_0.8_Sc_0.2_Co_2_O_4_	YSZ	Ni–YSZ	30.2	19.6	10.2	5.3	3.5		
(Mn_0.6_Mg_0.4_)_0.8_Sc_0.2_Co_2_O_4_–Ce	YSZ	Ni–YSZ	12.1	5.8	4.6	3.4	2.5		
(Mn_0.6_Mg_0.4_)_0.8_Sc_0.2_Co_2_O_4_–Pr	YSZ	Ni–YSZ	22.9	15.7	10.2	5.5	4.5		
NiCo_2_O_4_	ScCeSZ ^6^	Ni–YSZ	36.9	/	/	/	/	2021	[60]
CuBi_2_O_4_–GDC ^7^	YSZ	Ni–YSZ	0.135	0.104	0.083	0.067	0.057	2021	[66]
Ni_0.2_Fe_0.8_Co_2_O_4_	YSZ	Ni–YSZ	73.5	46.7	21.9	10.1	6.2	2021	[67]
Mg_0.4_NiMn_1.6_O_4_–YSZ	YSZ	Ni–YSZ	25	12	6	4	3	2021	[54]
Pr_2_CuO_4_	YSZ	Ni–YSZ	110	102	94.5	88	79	2022	[68]
Pr_2_CuO_4_–Pr_6_O_11_	YSZ	Ni–YSZ	42.5	41	39.8	36.9	30.2		
Mn_1.5_Cr_1.5_O_4_–GDC	YSZ	Ni–YSZ	0.032	0.021	0.015	0.009	0.005	2023	[58]
Mn_1.5_Cr_1.35_Mg_0.15_O_4_–GDC	YSZ	Ni–YSZ	0.168	0.12	0.079	0.048	0.03		
(Fe_0.2_Mn_0.2_Co_0.2_Ni_0.2_Zn_0.2_)_3_O_4_–GDC	YSZ	Ni–YSZ	3.19	2.48	1.72	1.41	0.09	2023	[69]
CuMn_1.5_Ni_0.5_O_4_–GDC	YSZ	Ni–YSZ	91.2	87.5	80.2	77.8	70	2023	[70]
Mg_0.4_Ni_1.4_Mn_1.2_O_4_–GDC	YSZ	Ni–YSZ	68	28	13	8	6	2023	[71]
(Mg_0.2_Fe_0.2_Co_0.2_Ni_0.2_Cu_0.2_)Fe_2_O_4_	YSZ	Ni–YSZ	1.14	0.73	0.33	0.19	0.147	2024	[59]
Ni(Fe_0.2_Mn_0.2_Co_0.2_Cr_0.2_Ni_0.2_)_2_O_4_	YSZ	Ni–YSZ	1.7	1.3	0.9	0.5	0.38	2024	[57]

^1^ YSZ: Y_0.08_Zr_0.92_O_1.95_ (10.8 × 10^−6^ K^−1^) [72]; ^2^ SSZ: 10 mol% Sc_2_O_3_ stabilized ZrO_2_ (11.1 × 10^−6^ K^−1^) [73]; ^3^ LSGM: La_0.9_Sr_0.1_Ga_0.8_Mg_0.2_O_3–δ_ (11.3 × 10^−6^ K^−1^) [74]; ^4^ SDC: Sm_0.1_Ce_0.9_O_1.95_ (12.6 × 10^−6^ K^−1^) [75]; ^5^ ScSZ: Sc_0.1_Zr_0.89_Ce_0.01_O_1.95_; ^6^ ScCeSZ: (Sc_2_O_3_)_0.1_(CeO_2_)_0.01_(ZrO_2_)_0.89_; ^7^ GDC: Gd_0.1_Ce_0.9_O_1.95_ (13.5 × 10^−6^ K^−1^) [76].

**Table 2:** The thermal expansion coefficient of different spinel oxides as reported in the literature.

Components			TEC (K^−1^)	Year	Reference
Air Electrode	Electrolyte	Fuel Electrode			
Mn_1.5_Co_1.5_O_4_	YSZ	Ni–YSZ	11.7 × 10^−6^	2011	[78]
CuCo_2_O_4_	SSZ	Ni–SSZ	11.76 × 10^−6^	2016	[73]
Cu_1.4_Mn_1.6_O_4_	SSZ	Ni–SSZ	12.1 × 10^−6^	2016	[62]
CoFe_2_O_4_	LSGM	Ni–SDC	13.4 × 10^−6^	2019	[63]
NiFe_2_O_4_	LSGM	Ni–SDC	12.5 × 10^−6^		
CuFe_2_O_4_	LSGM	Ni–SDC	12.1 × 10^−6^		
FeCo_2_O_4_–SDC	SDC	Ni–SDC	13.4 × 10^−6^	2020	[77]
MnCo_2_O_4_	YSZ	Ni–YSZ	14.7 × 10^−6^	2021	[65]
(Mn_0.6_Mg_0.4_)_0.8_Sc_0.2_Co_2_O_4_–Pr	YSZ	Ni–YSZ	13.9 × 10^−6^		
Ni_0.2_Fe_0.8_Co_2_O_4_	YSZ	Ni–YSZ	13.8 × 10^−6^	2021	[67]
NiMn_2_O_4_	YSZ	Ni–YSZ	9.6 × 10^−6^	2021	[54]
Mg_0.2_NiMn_1.8_O_4_	YSZ	Ni–YSZ	10.1 × 10^−6^		
Mg_0.4_NiMn_1.6_O_4_	YSZ	Ni–YSZ	10.6 × 10^−6^		
Mg_0.6_NiMn_1.4_O_4_	YSZ	Ni–YSZ	11 × 10^−6^		
Mg_0.8_NiMn_1.2_O_4_	YSZ	Ni–YSZ	11.7 × 10^−6^		
Zn_0.58_Co_2.42_O_4_	BZCY ^1^	Ni–BZCY	15.3 × 10^−6^	2022	[56]
Fe_0.6_Mn_0.6_Co_0.6_Ni_0.6_Cr_0.6_O_4_	BZCY	Ni–BZCY	10.2 × 10^−6^	2022	[8]
Pr_2_CuO_4_	YSZ	Ni–YSZ	11.5 × 10^−6^	2022	[68]
Pr_2_CuO_4_–Pr_6_O_11_	YSZ	Ni–YSZ	13.97 × 10^−6^		
CuMn_1.5_Ni_0.5_O_4_	YSZ	Ni–YSZ	11.94 × 10^−6^	2023	[70]
CuMn_1.5_Ni_0.5_O_4_–GDC	YSZ	Ni–YSZ	12.25 × 10^−6^		
Mg_0.4_NiMn_1.6_O_4_	YSZ	Ni–YSZ	11.4 × 10^−6^	2023	[71]
Mg_0.4_Ni_1.2_Mn_1.4_O_4_	YSZ	Ni–YSZ	12.3 × 10^−6^		
Mg_0.4_Ni_1.4_Mn_1.2_O_4_	YSZ	Ni–YSZ	13.3 × 10^−6^		
Mg_0.4_Ni_1.6_MnO_4_	YSZ	Ni–YSZ	14.2 × 10^−6^		
Mg_0.4_Ni_1.8_Mn_0.8_O_4_	YSZ	Ni–YSZ	14.7 × 10^−6^		

^1^ BZCY: BaCe_0.7_Zr_0.1_Y_0.2_O_3−δ_. (10.1 × 10^−6^ K^−1^) [79].

**Table 3:** The polarization resistance of different spinel oxides as reported in the literature.

Components			R_p_ (Ω cm^2^)					Year	Reference
Air Electrode	Electrolyte	Fuel Electrode	800 °C	750 °C	700 °C	650 °C	600 °C		
Mn_2_CoO_4_	YSZ	Ni–YSZ	1.06	/	5.17	/	/	2013	[61]
Mn_1.5_Co_1.5_O_4_	YSZ	Ni–YSZ	0.71	1.11	2.54	7.52	/		
MnCo_2_O_4_	YSZ	Ni–YSZ	2.46	/	7.17	/	/		
CuCo_2_O_4_	SSZ	Ni–SSZ	0.122	0.263	0.408	0.859	/	2016	[73]
Cu_1.4_Mn_1.6_O_4_	SSZ	Ni–SSZ	0.143	0.317	0.665	1.477	/	2016	[62]
CuCo_2_O_4_–SSZ	SSZ	Ni–SSZ	0.087	0.21	0.253	0.48	/	2017	[80]
CoFe_2_O_4_	LSGM	Ni–SDC	1.45	2.17	3.71	/	/	2019	[63]
NiFe_2_O_4_	LSGM	Ni–SDC	1.56	2.02	3.27	/	/		
CuFe_2_O_4_	LSGM	Ni–SDC	0.37	0.58	1.06	/	/		
Mn_1.3_Co_1.3_Cu_0.4_O_4_–ScSZ	ScSZ	Ni–YSZ	0.03	0.06	0.2	0.57	/	2019	[64]
MnCo_2_O_4_	YSZ	Ni–YSZ	/	1.09	2.3	4.2	8.9	2021	[65]
(Mn_0.6_Mg_0.4_)_0.8_Sc_0.2_Co_2_O_4_	YSZ	Ni–YSZ	/	/	1.02	1.98	3.8		
(Mn_0.6_Mg_0.4_)_0.8_Sc_0.2_Co_2_O_4_–Ce	YSZ	Ni–YSZ	/	0.24	0.55	1.28	3.3		
(Mn_0.6_Mg_0.4_)_0.8_Sc_0.2_Co_2_O_4_–Pr	YSZ	Ni–YSZ	/	0.11	0.25	0.58	1.3		
NiCo_2_O_4_	ScCeSZ	Ni–ScSZ	0.16	0.24	0.42	0.9	/	2021	[60]
CuBi_2_O_4_–GDC	YSZ	Ni–YSZ	/	/	0.58	1.62	5.59	2021	[66]
Ni_0.2_Fe_0.8_Co_2_O_4_–GDC	YSZ	Ni–YSZ	0.16	0.44	0.97	2.38		2021	[67]
Mg_0.4_NiMn_1.6_O_4_–YSZ	YSZ	Ni–YSZ	0.23	0.42	0.9	2.2	6.5	2021	[54]
Mn_1.3_Co_1.3_Cu_0.4_O_4_–ScSZ	SSZ	Ni–YSZ	0.05	0.13	0.32	1.3	/	2022	[51]
Pr_2_CuO_4_	YSZ	Ni–YSZ	/	/	0.21	/	/	2022	[68]
Pr_2_CuO_4_–Pr_6_O_11_	YSZ	Ni–YSZ	/	/	0.069	/	/		
Zn_0.58_Co_2.42_O_4_	BZCY	Ni–BZCY	/	/	0.0312	0.095	0.223	2022	[56]
Fe_0.6_Mn_0.6_Co_0.6_Ni_0.6_Cr_0.6_O_4_	BZCY	Ni–BZCY	/	/	0.057	0.15	0.45	2022	[8]
Mn_1.6_Cu_1.4_O_4_	BZCY	Ni–BZCY	/	/	0.1	0.3	0.89		
CoFe_1.9_Li_0.1_O_4_	BZCY	Ni–BZCY	/	/	0.035	0.11	0.19	2022	[81]
CoFe_2_O_4_	BZCY	Ni–BZCY	/	/	0.075	0.18	0.31		
Mn_1.5_Cr_1.5_O_4_–GDC	YSZ	Ni–YSZ	0.55	1.15	2.56	5.22	/	2023	[58]
Mn_1.5_Cr_1.35_Mg_0.15_O_4_–GDC	YSZ	Ni–YSZ	0.33	0.68	1.45	2.99	/		
(Fe_0.2_Mn_0.2_Co_0.2_Ni_0.2_Zn_0.2_)_3_O_4_–GDC	YSZ	Ni–YSZ	0.018	0.049	0.123	0.225	/	2023	[69]
CuMn_1.5_Ni_0.5_O_4_–GDC	YSZ	Ni–YSZ	0.043	0.123	0.43	1.83	6.21	2023	[70]
Mg_0.4_Ni_1.4_Mn_1.2_O_4_–GDC	YSZ	Ni–YSZ	0.25	0.45	0.71	1.51	3.56	2023	[71]
(Mg_0.2_Fe_0.2_Co_0.2_Ni_0.2_Cu_0.2_)Fe_2_O_4_	YSZ	Ni–YSZ	1.17	1.75	2.6	3.92	/	2024	[59]
Ni(Fe_0.2_Mn_0.2_Co_0.2_Cr_0.2_Ni_0.2_)_2_O_4_	YSZ	Ni–YSZ	0.63	1.23	2.52	3.63	/	2024	[57]
MnCo_1.9_Sb_0.1_O_4_–BZY	BZCY	Ni–BZCY	/	/	0.044	0.067	0.17	2024	[82]
MnCo_2_O_4_–BZY	BZCY	Ni–BZCY	/	/	0.094	0.23	0.53		
(Mg_0.2_Fe_0.2_Co_0.2_Ni_0.2_Cu_0.2_)Fe_2_O_4_–GDC	YSZ	Ni–YSZ	0.12	0.14	0.25	0.43	/	2025	[83]

**Table 4:** The Peak power density of different spinel oxides as reported in the literature.

Components			PPD (mW cm^−2^)					Year	Reference
Air Electrode	Electrolyte	Fuel Electrode	800 °C	750 °C	700 °C	650 °C	600 °C		
Mn_2_CoO_4_	YSZ	Ni–YSZ	386	216	100	/	/	2013	[61]
Mn_1.5_Co_1.5_O_4_	YSZ	Ni–YSZ	386	248	139	57	/		
MnCo_2_O_4_	YSZ	Ni–YSZ	160	137	59	/	/		
CuCo_2_O_4_	SSZ	Ni–SSZ	972	800	620	367	/	2016	[73]
Cu_1.4_Mn_1.6_O_4_	SSZ	Ni–SSZ	1076	809	512	301	/	2016	[62]
CuCo_2_O_4_–SSZ	SSZ	Ni–SSZ	1136	861	669	462	/	2017	[80]
CoFe_2_O_4_	LSGM	Ni–SDC	293	205	133	55	/	2019	[63]
NiFe_2_O_4_	LSGM	Ni–SDC	277	197	125	51	/		
CuFe_2_O_4_	LSGM	Ni–SDC	326	230	149	93	/		
Mn_1.3_Co_1.3_Cu_0.4_O_4_–ScSZ	ScSZ	Ni–YSZ	1920	1740	1380	/	/	2019	[64]
MnCo_2_O_4_	YSZ	Ni–YSZ	810.5	486	258.7	187	/	2021	[65]
(Mn_0.6_Mg_0.4_)_0.8_Sc_0.2_Co_2_O_4_	YSZ	Ni–YSZ	951.6	648.3	379.3	194.1	/		
(Mn_0.6_Mg_0.4_)_0.8_Sc_0.2_Co_2_O_4_–Pr	YSZ	Ni–YSZ	1311.4	1144.1	816.6	572.5	/		
NiCo_2_O_4_	ScCeSZ	Ni–ScSZ	1040	730	300	/	/	2021	[60]
CuBi_2_O_4_–GDC	YSZ	Ni–YSZ	/	507	326	192	110	2021	[66]
Ni_0.2_Fe_0.8_Co_2_O_4_–GDC	YSZ	Ni–YSZ	1930	1380	1020	590	250	2021	[67]
Mg_0.4_NiMn_1.6_O_4_–YSZ	YSZ	Ni–YSZ	526	486	416	348	218	2021	[54]
Mn_1.3_Co_1.3_Cu_0.4_O_4_–ScSZ	SSZ	Ni–YSZ	/	2110	1620	1000	/	2022	[51]
Pr_2_CuO_4_	YSZ	Ni–YSZ	/	/	680	402	197	2022	[68]
Pr_2_CuO_4_–Pr_6_O_11_	YSZ	Ni–YSZ	/	/	1070	700	326		
Zn_0.58_Co_2.42_O_4_	BZCY	Ni–BZCY	/	/	1603	1235	822	2022	[56]
Fe_0.6_Mn_0.6_Co_0.6_Ni_0.6_Cr_0.6_O_4_	BZCY	Ni–BZCY	/	/	1052	713	453	2022	[8]
Mn_1.6_Cu_1.4_O_4_	BZCY	Ni–BZCY	/	/	613	375	201		
CoFe_1.9_Li_0.1_O_4_	BZCY	Ni–BZCY	/	/	1052	751	503	2022	[81]
CoFe_2_O_4_	BZCY	Ni–BZCY	/	/	649	443	282		
Mn_1.5_Cr_1.5_O_4_–GDC	YSZ	Ni–YSZ	877	731	556	399	/	2023	[58]
Mn_1.5_Cr_1.35_Mg_0.15_O_4_–GDC	YSZ	Ni–YSZ	976	814	624	413	/		
(Fe_0.2_Mn_0.2_Co_0.2_Ni_0.2_Zn_0.2_)_3_O_4_–GDC	YSZ	Ni–YSZ	1080	920	750	550	/	2023	[69]
CuMn_1.5_Ni_0.5_O_4_–GDC	YSZ	Ni–YSZ	1342.4	1076.3	646.3	309.9	164.1	2023	[70]
Mg_0.4_Ni_1.4_Mn_1.2_O_4_–GDC	YSZ	Ni–YSZ	2020	1520	1230	720	340	2023	[71]
(Mg_0.2_Fe_0.2_Co_0.2_Ni_0.2_Cu_0.2_)Fe_2_O_4_	YSZ	Ni–YSZ	787.15	525	287	149	/	2024	[59]
Ni(Fe_0.2_Mn_0.2_Co_0.2_Cr_0.2_Ni_0.2_)_2_O_4_	YSZ	Ni–YSZ	583	435	290	172	/	2024	[57]
MnCo_1.9_Sb_0.1_O_4_–BZY	BZCY	Ni–BZCY	/	/	1380	981	673	2024	[82]
MnCo_2_O_4_–BZY	BZCY	Ni–BZCY	/	/	836	459	257		
(Mg_0.2_Fe_0.2_Co_0.2_Ni_0.2_Cu_0.2_)Fe_2_O_4_–GDC	YSZ	Ni–YSZ	809.64	617.74	410.8	220.09	/	2025	[83]

## Data Availability

The original contributions presented in this study are included in the article. Further inquiries can be directed to the corresponding author.
